# Factors Influencing Turnover Intention among Nurses and Midwives in Ghana

**DOI:** 10.1155/2022/4299702

**Published:** 2022-11-16

**Authors:** Angelina Boatemaa Boateng, Douglas Aninng Opoku, Nana Kwame Ayisi-Boateng, Alhassan Sulemana, Aliyu Mohammed, Joseph Osarfo, Jonathan N. Hogarh

**Affiliations:** ^1^Department of Occupational and Environmental Health, School of Public Health, Kwame Nkrumah University of Science and Technology, Kumasi, Ghana; ^2^Allen Clinic, Family Healthcare Services, Kumasi, Ghana; ^3^Department of Medicine, School of Medicine and Dentistry, Kwame Nkrumah University of Science and Technology, Kumasi, Ghana; ^4^University Hospital, Kwame Nkrumah University of Science and Technology, Kumasi, Ghana; ^5^Department of Environmental Science, College of Science, Kwame Nkrumah University of Science and Technology, Kumasi, Ghana; ^6^Department of Epidemiology and Biostatistics, School of Public Health, Kwame Nkrumah University of Science and Technology, Kumasi, Ghana; ^7^Department of Community Medicine, School of Medicine, University of Health and Allied Health Science, Ho, Ghana

## Abstract

**Background:**

Nurse turnover intention, defined as a measure of nurses' desire to leave their positions, is a global public health issue with a grave impact on the healthcare workforce. However, literature on it is limited in sub-Saharan Africa, an at-risk region. This study aimed to determine the predictors of turnover intention among nursing staff at a tertiary hospital in Kumasi, Ghana.

**Methods:**

This was an institution-basedcross-sectional study conducted among 226 randomly selected nurses and midwives working at a tertiary healthcare center in Kumasi, Ghana. Data were collected by using a structured questionnaire. Significant predictors of turnover intention were analyzed by using multivariate logistic regression analysis. Adjusted odds ratio (AOR) with a 95% confidence interval (CI) and *p* value <0.05 was used.

**Results:**

The prevalence of turnover intention among study participants was 87.2% (197/226). About two-thirds (61.5%, 139/226) of the participants were exposed to a high level of workplace hazards. Management support (AOR = 3.09, 95% CI = 1.09–8.75), salary (AOR = 0.07, 95% CI = 0.01–0.46), inadequate number of staff on duty per shift (AOR = 3.36, 95% CI = 1.08–10.47) and participants' rank (AOR = 6.81, 95% CI = 1.18–39.16) were significantly associated with turnover intention.

**Conclusion:**

Overall, the turnover intention was high. Hence, there is a need for policymakers, health administrators, and nurse managers to implement strategies such as increasing staff strength, providing adequate support, incentives, and other forms of motivation for nurses and midwives to help reduce the rate of turnover intention.

## 1. Introduction

Globally, the increasing demand for healthcare workers, particularly amidst the outbreak of the 2019 coronavirus disease (COVID-19), calls for the need to retain the healthcare workforce. Nurses and midwives form a significant proportion of the hospital workforce. The World Health Organization (WHO) estimates that nurses and midwives constitute approximately 50% of healthcare workers [1]. Nurses and midwives have an important role to play in the achievement of the Agenda 2030 of the Sustainable Development Goal Three (SDG3) of “good health and wellbeing for all” [2]. To be able to achieve SDG3 by 2030, the WHO projects that about 9 million additional nurses and midwives are needed [1].

About half of the global shortage of healthcare workers is attributable to nurses and midwives [1]. This has been heightened by the COVID-19 pandemic with its associated high workload, frequency of nurse absenteeism, increased levels of burnout, and turnover intention [3–6]. Mosallam et al. defined turnover intention as “the final cognitive step leading to actual turnover and that it is the main factor impacting turnover” [7]. Studies have shown that nurses' and midwives' turnover intention range from 20.0 to 67.8% globally [8–11]. In a multinational study, factors such as inadequate staff and job satisfaction were identified to contribute significantly to turnover intention among nurses and midwives [12]. Similarly, in Italy, Sasso et al. reported that poor patient safety and emotional exhaustion were significantly associated with turnover intention among nurses [13]. In sub-Saharan Africa, a pooled prevalence of 50.7% of turnover intention has been reported in a systematic review and meta-analysis [9]. In Ethiopia, a study found that about 64.9% of nurses had turnover intention [14]. Lack of satisfaction with performance appraisal systems, low monthly income, high workload, and young age have been identified as significant predictors of turnover intention among nurses and midwives in sub-Saharan Africa [11, 15, 16].

In Ghana, up to 69.0% of nurses and midwives have been reported to have turnover intention in the future [17–20]. It has been suggested that such turnover intention could be reduced if organizational and nurse managers would adopt a transformational leadership style to inspire positive change [19]. Burnout and workplace violence have also been reported to have a significant effect on turnover intention among nurses [17, 20]. For instance, Opoku et al. found in their study that nurses that experienced high burnout at the workplace had about five times increased odds of turnover intention [20].

This study sought to determine the prevalence and key predictors of turnover intention among nurses and midwives working at a tertiary hospital in Kumasi, Ghana. This study also assessed the exposure levels of workplace hazards and their effect on turnover intention among this group of healthcare workers. One key thing is that studies conducted in Ghana about the subject failed to address the effect of exposure levels of workplace hazards (physical, chemical, mechanical, and psychological hazards) on turnover intention among nurses and midwives. This study, therefore, addresses this gap in the effect of exposure levels of workplace hazards on turnover intention among nurses and midwives in Ghana. The outcome of this study will provide essential data for policymakers in formulating interventions that will aim at retaining nurses and midwives on the job and also reducing exposure to workplace hazards. The ability to retain nurses and midwives in the job in Ghana will also aid in improving the quality of patient care and strengthening the healthcare system of the country.

## 2. Methods

### 2.1. Study Design, Setting, and Population

This cross-sectional study was conducted among nurses and midwives working at a tertiary healthcare facility in Kumasi, Ghana, from September 2021 to December 2021. The study site has over 4000 healthcare professionals with diverse expertise. Among the healthcare professionals working in the facility are 2289 registered nurses and midwives. The study population was all registered and certified nurses and midwives working at the hospital. These were staff that performed nursing care at the hospital. The eligibility criteria for the study were all registered nurses and midwives that had practiced for at least 12 months at the facility. All nurses and midwives that were on study, annual and sick leave at the time of the data collection were excluded from the study.

### 2.2. Sample Size Estimation and Sampling

The sample size was calculated using a baseline study conducted in Nigeria [15], which showed that 85.5% (*P*) of healthcare workers in Nigeria had turnover intention. Based on a 95.0% confidence interval (*Z*) and a 5% allowable margin of error (*E*), the sample size was calculated from the following equation: [21]

(1) $$\begin{array}{c} Sample\;size=\frac{Z^{2\;}\left(P\right)\left(1-P\right)}{E^{2}}. \end{array}$$

With a nonresponse rate of 20%, a total of two hundred and twenty-nine (229) participants were recruited for the study.

The study participants were selected by using a simple random sampling technique. They were nurses and midwives working in a tertiary healthcare center in Kumasi, Ghana. First, balloting was carried out to select six out of thirteen clinical departments at the hospital. The number of nurses and midwives selected from each department was determined using probability proportional to size. This was performed by dividing the number of nurses in each department by the number of nurses in all the departments and multiplying by the estimated sample size. The selection of study participants was conducted by using the lottery method. Unique identifiers were assigned to all the names of nurses and midwives in each department. The unique identifiers were put in a bowl and drawn one after the other until the estimated sample size for each department was obtained.

### 2.3. Data Collection Tools and Procedure

Data collection was conducted by using a structured pretested questionnaire using a pen-to-paper approach. A pretest was carried out on 20 nurses and midwives working in a different facility in Kumasi which was not part of the source population for the study. After the pretest, appropriate revisions were made to the data collection instrument. The questionnaire was made up of four major parts; demographic characteristics (age, sex, educational level, among others), workplace environment (management support, leadership style, years of practice, among others), exposure to occupational hazards (excessive workload, slips, falls, extreme temperature, among others), and turnover intention.

The turnover intention was estimated by asking participants if they intended to quit the job in the future with a “yes” or “no” response. The turnover intention was defined as having the intention to quit the job in the future. The level of exposure to workplace hazards was assessed by asking the study participants about seventeen (17) workplace hazards that healthcare workers are potentially exposed to at the workplace. The exposure to a particular hazard was scored ‘1'. The overall score for the total number of workplace hazards exposed was computed and expressed as a percentage. Study participants that were exposed to at least 70.0% of the hazards were categorized as having a potentially high level of exposure, while those that had less than 70.0% were categorized as having relatively low levels of exposure. The cutoff of the exposure level was determined at the discretion of the authors.

### 2.4. Data Management and Analysis

The data were exported to Stata version 16 for quality checks and analysis. Descriptive statistics such as frequencies, percentages, and means were presented using tables. Chi-square or Fischer's exact test was used to determine the relationship between demographic characteristics, workplace environment, occupational hazards, and turnover intention. Significant predictors of turnover intention were analyzed by using multivariate logistic regression analysis. Adjusted odds ratio (AOR) with a 95% confidence interval (CI) and *p* value <0.05 were used to identify significant predictors of turnover intention.

### 2.5. Ethical Considerations

This study was granted ethical approval by the Komfo Anokye Teaching Hospital Institutional Review Board (KATH-IRB) with reference number KATHIRB/AP/040/20 on the 16^th^ of September, 2021. All recruited study participants provided informed consent. Their privacy and confidentiality were ensured.

## 3. Results

### 3.1. Demographic Characteristics and Workplace Environment of Study Participants

Out of a total of 229 participants that were recruited, 226 of them returned the questionnaire filled (response rate = 98.7%). About 45.1% (102/226) of the participants were between the ages of 30 to 39 years. The mean age of study participants was 32.9 (±7.2) years, with a range of 22 to 59 years. About two-thirds (65.0%, 147/226) of the participants were married. A little over half (52.7%, 119/226) of the participants had a diploma as their highest level of education (Table 1).

Approximately 53.1% (120/226) of the participants had practiced for 1 to 5 years. About 37.2% (84/226) of the participants were staff nurses/midwives. About 31.9% (73/226) of the participants indicated they received adequate management support, while about 32.3% (73/226) of the participants indicated they liked the leadership style in the ward (Table 1).

### 3.2. Turnover Intention and Exposure to Workplace Hazards among Study Participants

Out of a total of 226 participants, about 87.2% (197/226) of participants had turnover intention. About two-thirds (61.5%, 139/226) of the participants were exposed to a high level of workplace hazards. The study participants indicated they were exposed to the following hazards at the workplace: slips, trips, and falls (81.9%), infections from patients (90.3%), excessive workload (73.9%), standing for prolonged periods (72.6%), poor work posture (73.5%), and manual lifting of patients (86.3%) (Table 2).

### 3.3. Predictors of Turnover Intention among Study Participants

After adjusting for all the significant variables in the multivariate analysis, participants' rank, salary, management support, and the number of staff on duty per shift were identified as significant predictors of turnover intention among study participants (Table 3).

The odds of senior staff nurse/midwife having turnover intention was more (AOR = 6.81, 95% CI = 1.18–39.16) compared to staff nurse/midwife. Similarly, participants that were taking monthly salary between Gh¢3000–4000 ($369–$492) had about 93% (AOR = 0.07, 95% CI = 0.01–0.46) reduced odds of having turnover intention compared to those that were on monthly salary between Gh¢1000–2000 ($123–$246).

Study participants that indicated that management support was inadequate were about three (3) times more (AOR = 3.09, 95% CI = 1.09–8.75) likely to have turnover intention compared to those that indicated management support was adequate. Study participants that indicated that the number of staff per shift was inadequate were about 3 times more (AOR = 3.36, 95% CI = 1.08–10.47) likely to have turnover intention compared to those that indicated the number of staff per shift was adequate.

## 4. Discussion

Turnover intention is a major public health challenge, especially in low-resource settings. This study was conducted to determine the key predictors of turnover intention among nurses and midwives working in a tertiary hospital in Kumasi, Ghana. The study also examined the exposure of nurses to workplace hazards. The prevalence of turnover intention was estimated at 87.2%, with low salary levels being the commonest reason for turnover intention. The significant predictors of turnover intention in the present study were management support, salary, inadequate number of staff per shift, and participants' rank. About 61.5% of the study participants were exposed to high levels of workplace hazards.

The prevalence of turnover intention (87.2%) in the present study was about twice higher than the prevalence rate (45.2%) previously reported in Ghana [19]. The prevalence rate in this present study is comparable to studies conducted in Saudi Arabia (94.0%) and Japan (74.1%) [22, 23]. However, our 87.2% prevalence of turnover intention differs from the 20.7% reported in the Philippines [24] and 24.8% in Egypt [16].

The study participants were found to have been exposed to workplace hazards such as slips, trips and falls, cuts, wounds, needle pricks, and injuries from sharp objects, infections from patients, excessive workload, standing for prolonged periods, poor work posture, and manual lifting of patients. The exposure of nurses and midwives to cuts, wounds, needle pricks, and injuries from sharps is not surprising due to their frequent use of sharp objects, such as needles, for injections and other sharp objects at the workplace [25]. This finding is comparable to a study in Saudi Arabia that reported that among healthcare workers, nurses (56.5%) were the most affected by sharp object injuries at the workplace [26]. A higher prevalence of needle stick injuries has also been reported among Iranian nurses in the workplace [27]. This puts them at higher risk of cuts, wounds, needle pricks, and injuries from sharps. Again, these sharps may have been exposed to infected body fluid and blood, which could serve as a medium of transmission of infectious diseases. The majority of the nurses and midwives in this study indicated exposure to infections from patients as one of the common workplace hazards. This could be explained by the study being conducted amidst the COVID-19 pandemic where the infection rate was high. The high exposure to infections from patients in this study underpins an initial report that, as of June 2020, more than 600 nurses had lost their lives due to COVID-19 infections in the world [28]. The 81.9% of the study participants that indicated exposure to slips, trips, and falls is also very disturbing, looking at their effects on sustaining injuries at the workplace [29]. In the univariate analysis, participants who were exposed to high levels of workplace hazards had about three times increased risk of turnover intention compared to those that were exposed to low levels of workplace hazards. However, this was insignificant after adjusting for other covariates. We recommend that hospital architecture and structures should aim at minimizing injury risk.

The present study identified nurses' salaries and management support as significant predictors of turnover intention. This is in line with other studies that reported lack of management support could influence turnover intention among nurses and midwives [30–32]. In a previous study, nurses and midwives with high salaries were also more likely to remain in the profession compared to colleagues with lower salaries [33, 34]. Participants in the present study that indicated that they received inadequate support from management were more likely to have turnover intention compared to those who received adequate support from management. This highlights the importance of management support at the workplace and how it influences nurses' and midwives' turnover intention. When nurses and midwives receive all the needed support, such as the provision of personal protective equipment and medical care policy for the nurse and family, they may feel motivated. Our findings also have implications for policymakers and employers to ensure that salary levels and other forms of remuneration of nursing staff in sub-Saharan Africa are comparable with the global market.

We observed that an inadequate number of nurses on duty predicted turnover intention among study participants. Participants that had inadequate number of staff per shift were more likely to have turnover intention compared to those that had adequate number of staff per shift. In Australia and California in the United States, mandatory nurse-to-patient ratios per shift are as follows: 1 : 4 on morning shifts, 1 : 5 on afternoon shifts, and 1 : 8 on night shifts. For the type of ward, the nurse-to-patient ratios are 1 : 5 for the general medical-surgical ward, 1 : 4 for the emergency ward, and 1 : 2 for the critical care unit [35, 36]. There are no such recommendations in Ghana and across Africa. Inadequate number of staff per shift means that the burden of workload will be increased. This may also increase the stress levels of the number of staff on duty. High stress among nurses has been reported to influence turnover intention among nursing professionals [37]. There is, therefore, the need to ensure that the number of nurses on duty per shift is adequate to reduce the burden of workload as well as enhance patient and staff safety [38].

We also observed that participants' rank was significantly associated with turnover intention among nurses and midwives. Principal nurses/midwives and senior staff nurses/midwives were more likely to have turnover intention compared to staff nurses/midwives. The findings in the present study are comparable to an earlier study that found a significant effect on nurses' rank on turnover intention [39]. In Ghana, staff nurses/midwives are younger and of lower rank compared to senior staff nurses/midwives and principal nurses/midwives who have attained that status through long service. The leadership positions at the workplace are given to those of higher rank. Taking up a leadership position could increase the workload and stress levels at the workplace, which equally affects turnover intention [38].

The outcome of the present study will help hospital administrators, nurse managers, and policymakers to set up strategies that will reduce the levels of exposure to workplace hazards. Again, the high turnover intention among nurses and midwives in the present study deepened the significance of providing incentives and adequate support to nurses and midwives and ensuring that there is adequate number of staff on duty, considering their influence on turnover intention.

## 5. Strengths and Limitations of the Study

The present study provides useful data in understanding the dynamics of turnover intention among nurses and midwives in Ghana and expanding on existing interventions to improve their working conditions in the country. This study provides data on the effect of the levels of exposure to workplace hazards on turnover intention, which had not been previously reported among nurses and midwives in Ghana. Another strength of this study is the high response rate which enhances the quality of the study data and the representativeness of the study population.

Despite the significant findings in this study, a few limitations were identified. First, the exclusion of nurses and midwives on study, annual and sick leaves from the study could affect the burden of turnover intention. Second, this was not a multicenter study, hence, limiting the power of generalization of the study findings. Third, it is worth noting the wide confidence intervals of some of the results. We recommend that caution should be taken in interpreting the results with wide confidence intervals.

## 6. Conclusion

Turnover intention is rife among this cohort of nurses and midwives. The most significant predictors of turnover intention were management support, salary, inadequate number of staff per shift, and participants' rank. Again, study participants were exposed to a high level of workplace hazards. There is a need to set up strategies such as increasing the staff strength and ensuring equitable distribution of nurses and midwives in all the departments, providing the needed resources such as personal protective equipment and incentives for nurses. Amidst a high shortage of healthcare staff, we recommend a concerted effort and institution of policies to mitigate turnover intention.

## Figures and Tables

**Table 1:** Demographic characteristics and workplace environment of study participants.

Variables	Total *n* (%)	Intention to quit *n*(%)	No intention to quit *n* (%)	*p* values
*Age group (years)*				0.448^a^
20–29	86 (38.1)	72 (83.7)	14 (16.3)	
30–39	102 (45.1)	90 (88.2)	12 (11.8)	
40+	38 (16.8)	35 (92.1)	3 (7.9)	
Mean age (±SD)	32.9 (±7.2)			
Age range	22–59			

*Sex*				0.051
Male	66 (29.2)	62 (93.9)	4 (6.1)	
Female	160 (70.8)	135 (84.4)	25 (15.6)	

*Relationship status*				0.606^a^
Single	76 (33.6)	64 (84.2)	12 (15.8)	
Married	147 (65.0)	130 (88.4)	17 (11.6)	
Cohabiting	3 (1.3)	3 (100.0)	0 (0.0)	

*Level of education*				0.109
Diploma	119 (52.7)	107 (89.9)	12 (10.1)	
First degree	89 (39.4)	77 (86.5)	12 (13.5)	
Postgraduate	18 (8.0)	13 (72.2)	5 (27.8)	

*Years of practice*				0.056^a^
1–5	120 (53.1)	103 (85.8)	17 (14.2)	
6–10	51 (22.6)	49 (96.1)	2 (3.9)	
11–15	35 (15.5)	27 (77.1)	8 (22.9)	
16+	20 (8.9)	18 (90.0)	2 (10.0)	

*Rank*				0.003^a^
Staff nurse/Midwife	84 (37.2)	73 (86.9)	11 (13.1)	
Senior staff nurse/Midwife	69 (30.5)	67 (97.1)	2 (2.9)	
Nursing/Midwifery officer	43 (19.0)	33 (76.7)	10 (23.3)	
Senior nursing/Midwifery officer	9 (4.0)	6 (66.7)	3 (33.3)	
Principal nursing/Midwifery officer	21 (9.3)	18 (85.7)	3 (14.3)	

*Salary (Gh¢)*				0.033
1000–2000	87 (38.5)	77 (88.5)	10 (11.5)	
2000–3000	105 (46.5)	95 (90.5)	10 (9.5)	
3000–4000	34 (15.0)	25 (73.5)	9 (26.5)	

*Management support*				<0.001
Adequate	72 (31.9)	53 (73.6)	19 (26.4)	
Inadequate	154 (68.1)	144 (93.5)	10 (6.5)	

*Leadership style at the ward*				<0.001
Like	73 (32.3)	52 (71.2)	21 (28.8)	
Dislike	153 (67.7)	145 (94.8)	8 (5.2)	

*Effect of work on the matrimonial home*				0.001
Yes	97 (42.9)	93 (95.9)	4 (4.1)	
No	129 (57.1)	104 (80.6)	25 (19.4)	

*Number of staff on duty per a shift*				<0.001
Adequate	53 (23.5)	37 (69.8)	16 (30.2)	
Inadequate	173 (76.6)	160 (92.5)	13 (7.5)	

NB: postgraduate includes masters, GHC 8.13: USD 1.00 per exchange rate in Ghana during the study period, SD : standard deviation, ^a^analyzed by using Fisher's exact test.

**Table 2:** Turnover intention and exposure to workplace hazards among study participants.

Variable	Frequency, *N* = 226	Percentage, %
*Turnover intention*		
Yes	197	87.2
No	29	12.8

*Reasons for turnover intention * ^*∗*^		
To seek greener pastures abroad	147	74.6
Salary is too small	190	96.4
Planned career change	62	31.5
Too much abuse	55	27.9
Poor condition of service	179	90.9
My physical health	42	21.3
Lack of respect from doctors	76	38.6
Lack of respect from supervisors	79	40.1

*Level of exposure to workplace hazard*		
Low	87	38.5
High	139	61.5

*Exposure to workplace hazards * ^*∗*^		
Slips, trips, and falls	185	81.9
Cuts, wounds, needle pricks, and injuries from sharp objectives	211	93.4
Radiation	45	19.9
Extreme temperature (cold/heat)	53	23.5
Electric shock	61	27.0
Infection from patients	204	90.3
Irritation from disinfectants	125	55.3
Direct contact with a contaminated specimen	98	43.4
Heat	64	28.3
Anaesthetic gas/agents	91	40.3
Excessive workload	167	73.9
Poor interpersonal relationship	99	43.8
Standing for prolonged periods	164	72.6
Chemical inhalation	60	26.5
Poor work posture	166	73.5
Manual lifting of patients	195	86.3
Assault (verbal abuse)	69	30.5

^*∗*^Multiple responses.

**Table 3:** Multivariate logistic regression analysis of the factors associated with turnover intention among study participants.

Variables	*Univariate*		*Multivariate*	
	Crude OR (95% CI)	*p* value	Adjusted OR (95% CI)	*p* value
*Rank*				
Staff nurse/midwife	1.00		1.00	
Senior staff nurse/midwife	5.05 (1.08–23.61)	0.040	6.81 (1.18–39.16)	0.032
Nursing/midwifery officer	0.50 (0.19–1.29)	0.149	0.63 (0.17–2.38)	0.494
Senior nursing/midwifery officer	0.30 (0.07–1.38)	0.123	1.25 (0.17–9.42)	0.829
Principal nursing/midwifery officer	0.90 (0.23–3.58)	0.886	11.79 (1.20–115.98)	0.034

*Salary (Gh¢)*				
1000–2000	1.00		1.00	
2000–3000	1.23 (0.49–3.12)	0.657	1.04 (0.29–3.67)	0.955
3000–4000	0.36 (0.13–0.99)	0.047	0.07 (0.01–0.46)	0.005

*Management support*				
Adequate	1.00		1.00	
Inadequate	5.16 (2.26–11.81)	<0.001	3.09 (1.09–8.75)	0.033

*Leadership style at the ward*				
Like	1.00		1.00	
Dislike	7.32 (3.06–17.54)	<0.001	3.17 (0.93–10.80)	0.065

*Effect of work on the matrimonial home*				
Yes	1.00		1.00	
No	0.18 (0.06–0.53)	0.002	0.40 (0.10–1.52)	0.179

*Number of staff on duty per shift*				
Adequate	1.00		1.00	
Inadequate	5.32 (2.36–12.02)	<0.001	3.36 (1.08–10.47)	0.037

*Level of exposure*				
Low	1.00		1.00	
High	3.60 (1.59–8.18)	0.002	1.68 (0.62–4.56)	0.304

NB: all significant variables were adjusted for in the multivariate analysis, OR : odds ratio, and CI : confidence interval.

## Data Availability

The datasets used for the analysis of this study are available upon reasonable request from the corresponding author.
